# Health-seeking behaviour of rural women regarding cervical cancer prevention in Namibia: A qualitative study

**DOI:** 10.4102/jphia.v17i1.1632

**Published:** 2026-04-29

**Authors:** Elizabeth K. Ndakukamo, Roswitha Mahalie, Panduleni Hailonga-Van Dijk

**Affiliations:** 1 Department of Preventative Health Sciences, Faculty of Health, Natural Resources and Applied Sciences, Namibia University of Science and Technology, Windhoek, Namibia; 2 Department of Public Health, Seahorse Research and Training Institute, Windhoek, Namibia

**Keywords:** cervical cancer, health-seeking behaviour, rural women, Namibia, screening barriers, qualitative study

## Abstract

**Background:**

Cervical cancer preventative services exist in Namibia; however, these services are not easily accessible to women living in rural areas, which leads to late presentation for cervical screening and preventable morbidity as well as mortality. Understanding the sociocultural and systemic impediments to health-seeking behaviour is imperative for designing responsive interventions.

**Aim:**

To explore the perceptions, experiences and health-seeking behaviour of rural women in Namibia regarding cervical cancer prevention.

**Setting:**

Six rural constituencies in the Ohangwena and Kavango West regions of Namibia were covered in the study.

**Methods:**

An exploratory, descriptive qualitative design was employed. Moreover, data were collected through five focus group discussions with 40 women aged 20–65 years, selected purposively. Discussions were audio-recorded, transcribed, translated into English and analysed thematically using ATLAS.ti 23.

**Results:**

Six themes emerged: (1) low awareness and misinformation about cervical cancer; (2) structural impediments, including extended distances; (3) transport costs as well as overcrowding of health facilities; (4) negative healthcare experiences, including poor communication; (5) lack of confidentiality; and (6) cultural norms limiting open dialogue and autonomy. Motivators for screening included encouragement from health workers, self-motivation and perceived risk.

**Conclusion:**

Sociocultural and systemic impediments significantly influence rural women’s engagement in cervical cancer prevention activities.

**Contribution:**

This study provides context-specific insights into the health-seeking behaviour of rural women in Namibia, informing policy and practice for more equitable cervical cancer prevention.

## Introduction

Cervical cancer is one of the most preventable types of cancer, yet it continues to pose a disproportionate challenge for women in low- and middle-income countries (LMICs). Globally, more than 90% of cervical cancer deaths occur in LMICs, despite the availability of effective preventive measures such as the human papillomavirus (HPV) vaccination as well as regular screening.^1^ The prevalence of cervical cancer screening in sub-Saharan Africa (SSA) is low, at 10.29%.^2^ Unlike numerous other cancers, cervical cancer presents a unique opportunity for elimination, as affirmed by the World Health Organization (WHO), which launched a global strategy to eliminate cervical cancer as a public health concern.^3^ It is imperative to note that numerous countries in SSA, including Namibia, have yet to realise the full benefits of these interventions because of complex socio-economic, cultural and systemic impediments.^4,5^

Namibia is among the countries classified by the WHO as having a high cervical cancer burden, with an age-standardised incidence rate exceeding 30 cases per 100 000 women annually, substantially higher than the global average of 13.3 cases per 100 000 women per year.^6^ The 5-year survival rate ranges from 45% to 55%, below the global average of 66%, reflecting late presentation as well as limited access to timely diagnosis and treatment.^6^ Despite the endorsements of the Namibian National Cervical Cancer Screening Policy Guidelines in 2018 by the Namibia Ministry of Health and Social Services (MoHSS), which include the use of Visual Inspection with Acetic Acid (VIA) and Pap smear screening, service uptake remains low, especially in rural areas.^2^ These challenges are compounded by health system constraints such as staff shortages, supply chain inconsistencies and weak referral pathways.^7^ At the individual and community levels, additional barriers, such as limited education, fear, stigma, low risk perception and prevailing gender norms, continue to hinder women’s participation in preventive care.^8,9^

While awareness about cervical cancer is relatively high among women in Namibia, being aware does not equate with health-seeking behaviour.^10^ Less than half of women who are aware of screening undergo it, suggesting that knowledge alone cannot prompt preventive action.^7^ Impediments to cervical cancer screening are sophisticated and interconnected. Firstly, sociocultural expectations often dictate that household and childcare responsibilities take precedence over personal health. Consequently, even when women are aware of recommended actions, they may postpone screening to fulfil familial obligations. Secondly, in many male-headed households, women may require spousal permission or access to financial resources before they can utilise health services. Moreover, uptake of preventive services, such as cervical cancer screening, is constrained by cultural norms of modesty, reluctance to expose intimate body parts to male health workers and the belief that care is only sought when symptoms are present. In addition, there is a reliance on traditional healers for initial care. The health system itself presents barriers, including long travel distances to clinics, inconsistent availability of screening services, shortages of trained personnel and occasional lack of necessary supplies. These factors create a complex environment that discourages timely health-seeking behaviour.

A growing body of literature from SSA reveals that women’s decisions to seek cervical cancer prevention services are embedded in broader socio-ecological systems; at the macro level, national health policies, funding priorities and cultural norms; at the meso level, community beliefs, health delivery systems and social networks; at the micro level, household decision-making dynamics and spousal support; and at the individual level, personal beliefs, knowledge and perceived vulnerability.^11^ Studies from Tanzania,^12^ Kenya^13^ and Malawi^14^ indicate that fear of stigma, perceived invulnerability, a lack of partner support, as well as mistrust in health systems contribute to delayed or forfeited screening, even when services are physically accessible. However, much of the research in Namibia has concentrated on quantitative epidemiological data as well as service coverage indicators, neglecting a critical gap in understanding women’s lived experiences, particularly in rural settings.^15,16,17^ A population-based study in SSA underscores urban–rural disparities in screening uptake in Namibia, with 23.8% of urban women screened, compared to only 14.2% of rural women; however, the study failed to explain the contextual drivers of these gaps.^18^ As a result, health programmes may lack the cultural relevance and local legitimacy needed to improve engagement with services. Therefore, this study seeks to explore the healthcare-seeking behaviour of rural women in Namibia regarding cervical cancer prevention. In particular, it aims to document their perceptions of cervical cancer as well as its risks, describe impediments and facilitators to preventive care, and explain how individual, familial and community-level factors intersect to influence their decisions. Through this exploration, the research intends to contribute to an evidence-based study that may inform culturally appropriate interventions as well as advance Namibia’s progress toward the WHO’s goal of cervical cancer elimination.

### Theoretical framework

This study is underpinned by the socio-ecological model of health behaviour, which emphasises that seeking healthcare is not determined solely by individual knowledge or attitudes; rather, it results from the dynamic interplay of multiple levels of influence.^11^ At the individual level, women’s perceptions of cervical cancer risk, awareness of preventive measures and personal attitudes shape their intentions to seek care. Interpersonal factors, such as spousal and family support, community expectations and peer influence, can either facilitate or constrain engagement with screening as well as vaccination services. At the organisational and community level, accessibility to health facilities, availability of trained providers and trust in health systems fulfil a critical role. Finally, structural and policy environments, including national health priorities, resource distribution, gaps in policy implementation, and cultural norms, create broader conditions that shape individual choices.

## Research method and design

### Study design and setting

This study employed an exploratory, descriptive qualitative design to explore rural women’s perceptions, experiences and behaviours related to seeking cervical cancer preventive care. This design was adopted to provide an in-depth understanding of subjective meanings and contextual factors shaping women’s health-seeking behaviour. The study was conducted in the Ohangwena and Kavango West regions of Namibia. Regional cancer registry reports (2006–2009) indicated that the Ohangwena and Kavango West regions had a particularly high proportion of female cancers (up to 35.9% of reported reproductive cancers in that period), while recent local studies in Ohangwena and Kavango West reported low screening uptake, as well as multiple impediments to prevention.^19^ The primary ethnic groups are the Ovambo and Kavango, each with distinct cultural practices, in particular regarding health and gender roles.

### Study population and sampling method

Although reproductive age is commonly defined as 15–49 years, this study focused on women aged 18–49 years, consistent with ethical considerations for adult participation. Purposive sampling was applied to identify participants who could provide rich and relevant information in the Ohangwena and Kavango West regions. Furthermore, the two regions were purposively selected to represent rural settings with differing access levels to cervical cancer prevention services. Within these regions, constituencies (12 in Ohangwena and 8 in Kavango West) were identified, and health facilities within each constituency were selected as recruitment sites because of their routine care of women. Nurses working in reproductive health assisted in identifying eligible women who met the inclusion criteria and were willing to share their experiences. Women were approached if they were within the reproductive age range (18–49 years), lived in the study area and had some exposure to reproductive health services such as cervical cancer screening, HPV vaccination, antenatal care or family planning. Moreover, this process ensured that participants had firsthand insight into the impediments, perceptions and decision-making processes that influence health-seeking behaviour. The sample size was guided by data saturation, which was reached when additional interviews no longer yielded new themes or perspectives.

### Data collection

Data were collected over 2 weeks in August 2024. A semi-structured interview guide developed in English and translated into Oshikwanyama and Rukwangali was administered. A trained team comprising the principal researcher and four female research assistants (two per region) conducted the study. One research assistant moderated the discussions, employing a semi-structured interview guide, while the principal researcher drafted notes as well as managed the audio recordings. To ensure validity and depth, the second research assistant paraphrased and posed follow-up questions to clarify or expand on participants’ responses. It is imperative to note that both research assistants were local community health workers and were fluent in the local languages. Moreover, they were trained in qualitative data collection techniques. The interview guide was piloted in Engela (Ohangwena region) and Kapako (Kavango West region). This process allowed for the assessment of the cultural appropriateness and relevance of the questions, leading to minor revisions. Specifically, certain reproductive health terms were replaced with locally understood and culturally acceptable expressions to reduce discomfort and encourage open discussion. For example, the question ‘Have you ever been screened for cervical cancer?’ was revised to ‘Have you ever checked at the clinic for any problem in your cervix or womb?’ to align with local language use. Each focus group discussion (FGD) lasted approximately 90 min and was conducted in a private venue.

### Data analysis

Audio recordings were transcribed verbatim and translated into English. The transcripts were cross-verified against the original recordings by two independent language editors, one fluent in Oshikwanyama and the other in Rukwangali, to ensure accuracy and fidelity to participants’ narratives. Data were managed and analysed using ATLAS.ti version 23. An inductive thematic analysis approach was employed, with initial codes generated directly from the transcripts and subsequently organised into themes and sub-themes reflecting participants’ perspectives. Coding was conducted by the principal investigator, and the research team met regularly to review the coding process and resolve any inconsistencies through consensus. No discrepancies were identified.

### Ethical considerations

Ethical clearance was obtained from the Research Ethics Committee of the Faculty of Health, Natural Resources and Applied Sciences at the Namibia University of Science and Technology (NUST) (Ref: NUST/REC/2025/10) and from the Ministry of Health and Social Services (reference number 22/3/1/2). In addition, written informed consent was obtained from all participants prior to data collection. Confidentiality and anonymity were maintained throughout the study by assigning pseudonyms to participants and securely storing all audio files and transcripts on a password-encrypted device.

## Results

### Sociodemographic characteristics of participants

The study included 40 rural women, most of whom (47.5%) were between 40 years and 49 years of age. Nearly half (47.5%) had attained secondary education, while 42.5% had only primary or no formal education. The demographic characteristics of the participants are illustrated in Table 1.

**TABLE 1:** Sociodemographic characteristics of participants (*N* = 40).

Variables	Categories	*n*	%
Age (years)	18–29	5	12.5
	30–39	16	40.0
	40–49	19	47.5
Education	Primary or illiterate	17	42.5
	Secondary	19	47.5
	Tertiary	4	10.0
Employment status	Unemployed	24	60.0
	Employed or self employed	16	40.0
Marital status	Single	31	77.5
	Married	9	22.5
Number of children	None	4	10.0
	1–5	26	65.0
	6 and above	10	25.0
Screened	Yes	18	45.0
	No	22	55.0

### Emerging themes and sub-themes

The study analysis revealed six themes and associated sub-themes, as outlined in Table 2. These themes reflect the multidimensional nature of health-seeking behaviours among rural women as well as the sociocultural and systemic factors influencing their decisions. The direct quotes are presented as per the FGD, per region (Ohangwena [OH] and Kavango West [KW]) and participant number (P).

**TABLE 2:** Outline of themes and sub-themes.

Theme	Sub-theme
1. Knowledge and awareness of cervical cancer	1.1. Limited understanding of cervical cancer and its symptoms 1.2. Misconceptions and misinformation 1.3. Basic awareness of prevention
2. Barriers to cervical cancer screening	2.1. Structural and logistical barriers 2.2. Service delivery and health system issues 2.3. Age and eligibility confusion
3. Healthcare experiences and alternatives	3.1. Mixed perceptions of health workers 3.2. Use of traditional or spiritual healing alternatives
4. Autonomy and cultural influence	4.1. Women’s independence in decision-making 4.2. Cultural fears and stigma
5. Motivators for screening uptake	5.1. Health worker encouragement 5.2. Self-motivation and perceived risk
6. Community and institutional support needs	6.1. Need for local health education and outreach services 6.2. Emotional support from family and community 6.3. Health promotion and material needs

### Theme 1: Knowledge and awareness of cervical cancer

#### Sub-theme 1.1: Limited understanding of cervical cancer and its symptoms

Even though 45% of the participants were screened for cervical cancer, about 70% of the participants demonstrated a limited or inaccurate understanding of cervical cancer and its symptoms. Moreover, while a few participants vaguely associated the disease with genital wounds, others had never received any health education on the topic. A participant stated the following:

‘No, not really. Nevertheless, we suspect that wounds or sores in the private area might be a sign. However, we are not certain and would like more information.’ (FGD1, OH, P8)

The uncertainty revealed in participants’ responses underscores a knowledge gap and the need for basic health education on cervical cancer:

‘I have heard about it, but I am not sure what causes it.’ (FGD2, OH, P12)

‘It sounds like something that happens to older women only, not people like us, young ones.’ (FGD3, OH, P19)

#### Sub-theme 1.2: Misconceptions and misinformation

Several participants reported misinformation circulating in their communities. Moreover, cultural myths and rumours contributed to fear and avoidance of screening services. Several participants shared beliefs not grounded in scientific understanding, for example, the belief that screening procedures involve removing the womb, which discouraged participation, while others attributed transmission to uncircumcised men. A participant stated:

‘There are rumours about the womb being removed during the screening procedure; that is why people do not want to go to the hospital for screening.’ (FGD5, KW, P33)

Conversely, some participants reported misinformation that cervical cancer screening is rooted in fear and cultural myths, perpetuating stigma and deterring women from seeking assistance. A participant added the following:

‘Some people in our village say that the tools used to screen women are not clean, and sometimes they cut you during the procedure, and it is very painful.’ (FGD1, OH, P6)

#### Sub-theme 1.3: Basic awareness of prevention

A few participants demonstrated awareness of preventive methods, such as condom use; however, the understanding of how this relates to cervical cancer was vague. A participant indicated:

‘Yes, as long as you are using a condom, I do not think you will get cervical cancer.’ (FGD3, OH, P17)

‘I think it is caused by unprotected sex, so staying faithful to one partner can help prevent you from contracting the disease.’ (FGD2, OH, P16)

However, knowledge of HPV and its vaccination as a prevention method was lacking, as reflected by some participants:

‘I have never heard of HPV before. If there is a vaccine, why aren’t we receiving it here at the clinic? They tell us about screening for cancer in women, but not that one.’ (FGD2, OH, P16)

‘I just found out about HPV during this discussion. If the vaccine can protect us, everyone should be able to receive it, not only school-aged girls.’ (FGD5, KW, P33)

### Theme 2: Barriers to cervical cancer screening

#### Sub-theme 2.1: Structural and logistical barriers

Regarding barriers to cervical cancer screening, some participants indicated that they were not screened because they could not afford transport costs to health facilities, particularly those who lived in the inland, making access to health services challenging. Furthermore, inaccessibility of clinics because of distance or poor roads was noted as a limitation. Participants indicated the following:

‘Some of us have to walk long distances from our homes because there are no taxis in our area, so sometimes you sit at home and wait until the sickness is gone.’ (FGD3, OH, P24)

‘It is far, and there is no regular transport in my village. If the mobile clinic does not come, we cannot go and most of the time the mobile clinics take months to visit.’ (FGD4, KW, P26)

#### Sub-theme 2.2: Service delivery and health system issues

Overcrowding and long waiting times during cervical cancer screening campaigns left some participants unattended and discouraged future attempts. A participant stated:

‘I tried attending a screening campaign when I visited one of my uncles in town, but the crowd was too big, we waited all day and went home without being seen.’ (FGD2, OH, P11)

Conversely, participants also reported inconsistent service delivery, such as unavailable staff or poorly organised outreach visits for VIA, which further contributed to their dissatisfaction. A participant corroborated this:

‘Sometimes they tell us the machine is not working, and one time I went there because the doctor referred me because my periods were not stopping. I was told to come back another day. They took my number and never called.’ (FGD5, KW, P35)

#### Sub-theme 2.3: Age and eligibility confusion

Several participants were misinformed about the eligibility criteria based on age, leading to early disengagement:

‘Sometimes, it is because of age restrictions. I was once told I was too young, so I never returned.’ (FGD1, OH, P2)

### Theme 3: Healthcare experiences and alternatives

#### Sub-theme 3.1: Mixed perceptions of health workers

While some participants expressed trust in health workers, others described their encounters as discouraging and emotionally distressing. Moreover, they indicated that healthcare workers were often unapproachable, disrespectful or did not maintain patient confidentiality. A participant expressed the following:

‘Some nurses have unfriendly attitudes, shout, and do not maintain confidentiality. You know my sister: sometimes they treat you based on appearance and status, they think you are pretending to be sick.’ (FGD4, KW, P40)

This perception created a sense of fear and reluctance to return for future services. A participant recalled:

‘When I asked questions, the nurse ignored me and moved on to the next person. I felt embarrassed and discouraged.’ (FGD2, OH, P16)

#### Sub-theme 3.2: Use of traditional or spiritual healing alternatives

In the absence of trust in or access to formal health services, several participants reported turning to alternative sources of healing, such as churches and traditional healers. More importantly, these practices were often rooted in cultural beliefs and community norms, offering emotional comfort and perceived healing. Participants stated:

‘Some people visit revival churches and spiritual houses, others visit traditional healers for preventing diseases from getting worse.’ (FGD5, KW, P36)

‘At church, the pastor prays for us and gives us holy water. It feels safer.’ (FGD3, OH, P23)

### Theme 4: Autonomy and cultural influence

#### Sub-theme 4.1: Women’s independence in decision-making

Numerous participants expressed a strong sense of autonomy in making decisions related to their health in general, including whether to undergo cervical cancer screening. This sense appeared to be shaped by a growing confidence among participants to take control of their well-being. A participant explained:

‘The decision to seek health care is entirely ours because only we can make it ourselves.’ (FGD1, OH, P4)

While some participants felt empowered, others acknowledged that male partners still held influence over health decisions, either explicitly or implicitly. In contexts where male support was lacking, autonomy could be challenging to exercise. A participant noted:

‘I make my own decisions about my health, but it is not easy. People talk, and they say a woman who goes for such tests without her husband’s permission is disrespectful. Sometimes I keep quiet to avoid conflict.’ (FGD4, KW, P30)

#### Sub-theme 4.2: Cultural fears and stigma

Cultural fear remained a significant constraint to open discussions about reproductive health issues. Numerous participants feared the consequences of challenging patriarchal expectations. A participant admitted:

‘If someone hears you are being tested for cervical cancer, they may think you have been promiscuous. That kind of judgment stops many women from going, even when they want to.’ (FGD2, OH, P10)

Another patient reflected:

‘Some people say that if you go for screening, it means you are already sick or have been unfaithful. That makes women afraid because they do not want to be talked about in the community, also, there is silence around sexuality, and it is even worse when you have to say you have a problem “down there.”’ (FGD4, KW, P28)

In addition, the association of cervical cancer with promiscuity or unfaithfulness created further stigma. Some women worried that seeking screening might lead to accusations or mistrust from their partners. A participant expressed:

‘If you mention screening, he asks, “Why? What have you been doing?” So, you keep quiet.’ (FGD1, OH, P3)

### Theme 5: Motivators for screening uptake

#### Sub-theme 5.1: Health worker encouragement

Participants consistently cited recommendations from nurses during routine visits, such as antenatal care, postnatal consultations or immunisation appointments, as crucial motivators. A participant noted:

‘The nurses recommended I go for screening.’ (FGD5, KW, P35)

This encouragement from health workers enabled participants to demystify the procedure and reduced anxiety about the outcome. It also reinforced that screening was a routine and a crucial aspect of women’s health. A participant recalled:

‘It was during a clinic visit when the nurse said we should all get checked. That pushed me to go.’ (FGD2, OH, P14)

#### Sub-theme 5.2: Self-motivation and perceived risk

Beyond external encouragement, some women were motivated by personal concern as well as a desire to stay healthy. Numerous participants indicated that they took the initiative to be screened after learning about the dangers of cervical cancer or suspecting that they might be at risk because of their own or their partner’s behaviour. A participant stated:

‘Some of us take the initiative because we want to know our cervical health status.’ (FGD2, KW, P9)

This proactive attitude reflected a growing shift in health consciousness and personal responsibility. A participant expressed:

‘Nowadays, it is hard to trust a partner fully, so we decided to get screened just to be safe.’ (FGD4, KW, P16)

### Theme 6: Community and institutional support needs

#### Sub-theme 6.1: Need for local health education and outreach services

Participants repeatedly called for broader awareness initiatives to improve community knowledge and dispel myths about cervical cancer. They proposed practical outreach strategies such as radio announcements, school talks and women’s group meetings. A participant explained:

‘They should give announcements via local radio to alert people.’ (FGD3, OH, P20)

Another participant added:

‘They must come to the villages, not just town clinics. Many people here do not know about this disease.’ (FGD5, KW, P38)

#### Sub-theme 6.2: Emotional support from family and community

Some participants noted that their sisters, mothers or close friends motivated them to seek screening and offered reassurance during the process. A participant indicated:

‘Their encouragement helps us through the process.’ (FGD1, OH, P2)

Such social support eased fears and reduced stigma. In addition, sometimes, participants went in groups or accompanied one another to health facilities. A participant remarked:

‘When someone you trust goes with you, it makes the whole thing less scary.’ (FGD2, OH, P16)

#### Sub-theme 6.3: Health promotion and material needs

In addition to education and emotional support, participants expressed the need for practical resources to promote cervical health. These included hygiene items, free screening supplies, vaccinations and modern preventive technologies. As one participant emphasised:

‘Modern preventive treatments, such as injections, would help because we have nothing right now.’ (FGD3, OH, P20)

The lack of access to basic health materials was perceived as an impediment to prevention as well as maintaining general reproductive health. Another added:

‘Even soap or pads would make a difference. We are often told to be clean, but do not have what we need.’ (FGD5, KW, P33)

## Discussion

This study explored the health-seeking behaviours of rural Namibian women regarding cervical cancer prevention. The key findings revealed several interrelated themes: (1) knowledge and awareness of cervical cancer; (2) barriers to cervical cancer screening; (3) healthcare experiences and alternatives; (4) autonomy and cultural influence; (5) motivators for screening uptake; and (6) community and institutional support needs.

This study underscores the persistence of low awareness and misinformation as significant impediments to cervical cancer prevention among rural women, reflecting a tendency widely documented in SSA.^20,21^ While several participants demonstrated basic awareness associating cervical cancer with sexual behaviour or hygiene, this knowledge was often vague or distorted by myths, such as the belief that screening involved the removal of the womb. These misconceptions indicate a more profound absence of culturally relevant health education as well as the dominance of informal information channels, echoing studies from Ethiopia and Uganda, where health messaging failed to bridge the gap between biomedical knowledge and local beliefs.^22,23^

The findings further revealed substantial structural and logistical constraints that inhibit screening access. The constraints include transport costs, long distances to clinics and poorly coordinated outreach engagements, reflecting evidence from Zimbabwe and Ethiopia, where financial and geographic challenges reduced screening uptake.^24^ Confusion about eligibility, especially among younger women, illustrates a lack of consistent public health communication. More importantly, this ambiguity may lead to missed opportunities for early detection, particularly in regions where cervical cancer often presents late as a result of delayed care-seeking.

Engagements with healthcare workers emerged as a defining influence on women’s screening behaviour. At the same time, positive interactions could motivate action, and negative encounters, indicated by perceived disrespect, breaches of confidentiality or dismissiveness, discouraged return visits. These accounts resonate with findings from Kenya, where provider attitudes were a determinant of perceived service acceptability.^8,13^ When health systems reproduce hierarchical or judgemental dynamics, especially in reproductive health contexts, they reinforce stigma and silence, particularly in patriarchal rural settings.

In settings where formal health services were perceived as inaccessible or untrustworthy, women turned to traditional or spiritual avenues for comfort and meaning. This reliance does not merely reflect a rejection of biomedical care or delays timely engagement with formal health services; rather, it represents a search for support systems that align with local values and provide agency within a culturally relevant context. As observed in Tanzania, such dual engagement with traditional and clinical options underscores the need for health systems to acknowledge and respectfully integrate local beliefs.^20^

Despite systemic challenges, the study documented examples of agency and resilience. Several participants reported independently deciding to undergo screening, even when this decision conflicted with partner expectations or prevailing cultural norms. These expressions of autonomy indicate that participants are not passive recipients of health information but active navigators of constrained choices, a finding consistent with feminist health research in other patriarchal contexts.^25^ Risk perception, emotional survival and self-preservation motivate health-seeking in ways that formal interventions often overlook.

Healthcare provider encouragement during routine visits also played an important role in screening uptake. Antenatal and immunisation appointments offered practical opportunities to provide cervical cancer information, consistent with findings from Zambia and Nigeria showing that provider recommendation is one of the strongest predictors of screening uptake.^26,27^ Notably, self-motivation also featured prominently; several participants pursued screening not just because of awareness, but out of concern for their health in the context of relationship uncertainty. This suggests a shift from passive awareness to proactive engagement grounded in personal risk assessment.

Lastly, participants underscored the need for specific institutional and community-level support, recommending radio-based campaigns, the involvement of family and community leaders, as well as the urgent provision of hygiene materials and modern preventive options such as HPV vaccination. These findings indicate a broader understanding among participants that cervical cancer prevention is inseparable from the wider conditions of reproductive health. More importantly, the interlinkage of education, access, emotional support and material needs reflects a holistic perception of health that is increasingly supported in global health literature.^8,28^ These findings indicate that while knowledge and awareness are essential, they must be embedded within an enabling environment where health systems are accessible, respectful and culturally relevant. Without addressing the structural, interpersonal and cultural dimensions of health-seeking, cervical cancer prevention efforts are likely to remain inadequate, particularly in rural contexts, where trust in the health system is fragile and choices are often constrained.

### Strengths and limitations

A significant strength of this study is embedded in its use of a qualitative, community-based approach that allowed for an in-depth exploration of women’s lived experiences in underserved regions. Moreover, conducting focus group discussions in local languages further enhanced authenticity and participant comfort. It is imperative to note that the study is limited by its geographic scope, as findings may not be generalisable beyond the Ohangwena and Kavango West regions. Additionally, social desirability bias may have influenced responses, particularly on sensitive matters related to reproductive health and gender dynamics.

### Implications and recommendations

These findings hold significant implications for policy and practice. Firstly, cervical cancer education should be decentralised and embedded into routine community health platforms. Local radio services, churches and women’s groups should be strengthened to correct misinformation, reduce stigma and normalise screening. Secondly, health worker training needs to prioritise respectful care as well as communication, particularly in rural health facilities where trust is already fragile. Thirdly, logistical impediments such as transport, equipment availability and mobile outreach need to be addressed through targeted investment in rural health infrastructure. More importantly, engaging male partners and traditional leaders at the community level may assist in reducing cultural stigma and supporting women’s autonomy in health decisions. Finally, policy attention needs to extend beyond awareness to include access to essential materials such as hygiene products, self-sampling kits and HPV vaccines, as integral to reproductive health equity. Moreover, future research should evaluate the long-term impact of health education campaigns and explore how digital instruments can support screening uptake in remote areas. Ultimately, cervical cancer prevention must be framed not only as a medical priority but as a reproductive justice matter, one that demands sustained attention to the social, structural, as well as cultural dimensions of rural women’s health.

## Conclusion

This study demonstrated that rural Namibian women encountered complex impediments to cervical cancer prevention, including poor awareness, cultural constraints and structural challenges. These findings underscore the need for culturally grounded, gender-sensitive and community-oriented approaches to improve screening uptake. Addressing these barriers could significantly reduce cervical cancer morbidity as well as mortality among these rural populations.
